# Commentary: Complete Genome Sequence of 3-Chlorobenzoate-Degrading Bacterium *Cupriavidus necator* NH9 and Reclassification of the Strains of the Genera *Cupriavidus* and *Ralstonia* Based on Phylogenetic and Whole-Genome Sequence Analyses

**DOI:** 10.3389/fmicb.2019.02011

**Published:** 2019-08-28

**Authors:** Han Ming Gan

**Affiliations:** ^1^Centre for Integrative Ecology, School of Life and Environmental Sciences, Deakin University, Geelong, VIC, Australia; ^2^Deakin Genomics Centre, Deakin University, Geelong, VIC, Australia; ^3^School of Science, Monash University Malaysia, Petaling Jaya, Malaysia

**Keywords:** *Ralstonia*, *Burkholderiaceae*, *Cupriavidus*, phylogenomics, taxonomy

Moriuchi et al. reported a comprehensive reclassification of bacterial strains from the genera *Cupriavidus* and *Ralstonia* based on percentage of conserved proteins (POCP), average nucleotide identity (ANI), multilocus sequence analysis and 16S rRNA gene sequence. In the study, conflicting results were repeatedly observed for the taxonomic classification of strain PBA that was initially identified as *Ralstonia* sp. PBA based on 16S rRNA gene sequence (Gan et al., 2011b; Moriuchi et al., 2019).

Strain PBA was isolated as a co-culture with *Hydrogenophaga intermedia* PBC from textile wastewater a decade ago. The co-culture could grow on 4-aminobenzenesulfonate (4-ABS), a recalcitrant dye intermediate (Wagner and Reid, 1931), as the sole nitrogen, carbon, and sulfur source to a relatively high cell density (Gan et al., 2011b). In this syntrophic relationship, strain PBA is the sole provider of *p*-aminobenzoate, an essential vitamin required for the growth of *H. intermedia* PBC, the main 4-ABS degrader (Gan et al., 2011a, 2017). In light of new genomic resources, the initial taxonomic assignment of strain PBA has also been previously questioned by Kim and Gan (2017) given its closer phylogenetic affiliation to the genus *Cupriavidus* than to the genus *Ralstonia*. Unfortunately, both recent genome-based taxonomic classifications of strain PBA (Kim and Gan, 2017; Moriuchi et al., 2019) suffered from incomplete and biased taxon sampling (restricted mostly to members from the genus *Ralstonia* and *Cupriavidus*) that can result in the misinterpretation of evolutionary relationships (Heath et al., 2008). The taxonomic affiliation of strain PBA should be inferred from a comprehensive phylogenomic analysis that includes all genera with genome availability from the family Burkholderiaceae.

A total of 428 Burkholderiaceae (including strain PBA) and 15 non-Burkholderiaceae genome assemblies were obtained from the NCBI RefSeq database (accessed on 30th May 2019). The genomes were processed using two microbial phylogenomic analysis pipelines e.g., GToTree v1.2.1 (Lee, 2019) and PhylophlAN v0.99 (Segata et al., 2013) that identify single copy bacterial genes (GToTree: *n* = 203, Betaproteobacteria HMM set; PhylophIAN: *n* = 400) and produce concatenated protein alignment. Maximum likelihood tree construction from the protein alignments used IQTree v.1.6.8 with 1,000 ultrafast bootstrap replicates (Nguyen et al., 2014). In both phylogenomic trees, the *Ralstonia* and *Cupriavidus* clusters received maximal support and are sister taxa to the exclusion of strain PBA (Figures 1A,B). The updated phylogenomic placement of strain PBA in light of extensive taxon sampling precludes its genus assignment to the genus *Ralstonia* or *Cupriavidus* and suggests that it is a member of a hitherto undescribed genus within the family *Burkholderiaceae*. Within the Genome Taxonomy Database (Parks et al., 2018) that inferred standardized bacteria taxonomy from conserved proteins present in 143,512 bacterial genomes (GTDB release R04-RS89), strain PBA was still assigned to its own genus (g__AKCV01) despite an even more extensive taxon sampling of 4,378 genomes from the family *Burkholderiaceae* (https://gtdb.ecogenomic.org/tree?r=g__AKCV01 accessed on 1st August 2019).

**Figure 1:**
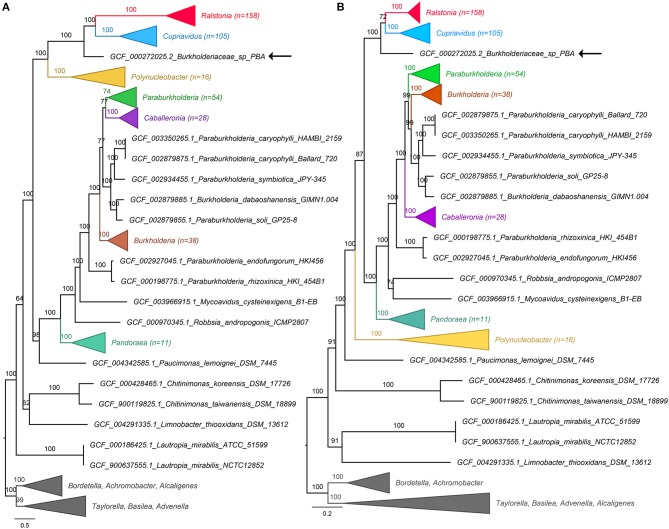
Genome-based phylogeny of Burkholderiaceae. Construction of the phylogenomic trees used protein alignments generated from **(A)** PhyloPhlAN and **(B)** GToTree. Numbers in brackets indicate number of taxa in the collapsed clades. GCF codes preceding the species names are RefSeq Assembly numbers. The positions of strain PBA in both trees are shown by black arrows. Branch lengths and labels indicate the number of substitutions per site and IQTree ultrafast bootstrap support values, respectively. The uncollapsed trees are available in the Zenodo database (http://doi.org/10.5281/zenodo.3258920).

Given the concordance observed from these independent analyses, the taxonomic assignment of strain PBA has been updated from *Ralstonia* sp. PBA to *Burkholderiaceae* sp. PBA in the NCBI database (Bioproject: PRJNA78957; BioSample: SAMN02471424) (Gan et al., 2012) pending future genus description. To facilitate future strain description and comparison, strain PBA has been deposited in the German Collection of Microorganisms and Cell Cultures GmbH (DSMZ) under the accession number DSM 106616. Furthermore, the concatenated alignments, uncollapsed phylogenomic trees and genome information are also made available in the Zenodo database (http://doi.org/10.5281/zenodo.3258920).

## Author Contributions

HG performed data analysis and wrote the manuscript.

### Conflict of Interest Statement

The author declares that the research was conducted in the absence of any commercial or financial relationships that could be construed as a potential conflict of interest.
